# Intestinal Digestion in Babies

**Published:** 1908-02-01

**Authors:** 


### Diseases of Children.

### INTESTINAL DIGESTION IN BABIES.

It is worth while considering some of the recent observations on the digestion and metabolism of food in the intestines of babies, naturally and artificially fed. The results are based on analysis and naked-eye examination of the urine and fæces, and on post-mortem investigation of the secretory and other functions of the intestines, liver and gall bladder, and pancreas. Zweifel and Korowin denied the existence of a diastasic ferment in the pancreas under three weeks of age. Moro found it present in seven out of ten babies, two of which had died at birth. Meconium contains a diastasic ferment, and the stools of early infancy can convert one-twentieth part of starch into sugar (Kerley, Mason and Craig). Gillet, as the result of post-mortem research, maintains that the pancreas contains no sugar-forming ferment during the first few months of life. Thus this question is still unsettled.

### The Stools in Infancy.

During illness all the functions of the pancreas may be affected. More reliable conclusions can be drawn from the study of the fæces than from post-mortem observations on extracts of the glands. The colour of the normal fæces varies with the diet, especially with the amount of fat in the milk. The stools of the breast-fed have an acrid, non-fœtid smell; an acid reaction, due to lactic acid; and a yellow colour, which turns green on exposure to air, on account of the conversion of bilirubin into biliverdin. Those of children fed on cow's milk are whitish or a pale yellow, and turn greyish-yellow or white on exposure. They have a strong smell and an alkaline reaction. The addition of carbohydrates to the diet renders the stools more yellow in colour, acid in reaction, and free from the smell of decomposition.

### A Note on the Bacteriology.

The processes of decomposition and fermentation of the intestinal contents are due to micro-organisms and intestinal juices. In infancy the absence of a putrefactive odour is partly due to rapid passage of food through the alimentary tract, for the stools will putrefy after excretion if kept for a long time; to the fact that milk does not usually undergo putrefaction, and a diet of milk and milk products lessens intestinal putrefactive processes; and to the fact that fermentation prevents putrefactive changes. Carbohydrates thus prevent putrefactive decomposition of casein. The organisms normally present in the intestines are the B. lactis aërogenes, B. coli communis and B. bifidus. All of them cause fermentation, but have no proteolytic or peptonising action. The acid reaction produced by fermentation in the intestinal contents prevents putrefaction. In pathological states a further and most important factor is introduced, namely, the secretion of mucus, a substance which is extraordinarily liable to putrefaction. It is to this that the foul smell of the stools in enteric disturbances is commonly due. In a previous article on gastric digestion it was shown that the secretion of hydrochloric acid is sometimes decreased or suppressed; that the acid acts as a valuable gastric disinfectant, inhibiting the growth of organisms; and that it is consequently a factor in the prevention of intestinal putrefaction. Putrefaction causes an alkaline reaction in the stools. In fermentation an acid is produced by the breaking up of carbohydrate, lactose in the case of the breast-fed child, other sugars and cereals perhaps in the bottle-fed. Escherich regards lactic acid as the normal product. Babinsky and others consider it a secondary formation, and regard succinic and acetic acids as the principal fermentation products of lactose.

### Intestinal Putrefaction.

Clinically, the important point is that intestinal putrefaction can be prevented by setting up acid fermentation and in this way destroying or inhibiting the growth of intestinal flora of putrefaction. Further, it is found that the stools are thereby hurried through the canal, for the fermentation increases peristalsis. Excessive or abnormal fermentation may be set up by too large or too frequent meals, or by unsuitable food, and may lead to excess of secretion, desquamation, and peristalsis. Thus, in fermentative dyspepsia there is much tympanites, from formation of gas, and numerous and frothy stools, with an acid reaction. Green stools have been ascribed by Pfeiffer to the excess of alkali in the gut converting the bilirubin into biliverdin. More probably, when evacuated as such, the colour is due to an oxidising ferment. Wernstedt has identified such a ferment in the intestinal mucus and leucocytes. The green colour is always associated with secretion of mucus, which he regards as the primary cause, the ferment action being secondary.

We know more about the reduction changes in bile pigment than about the oxidising of bilirubin. Fæces, containing hydrobilirubin, turn red when treated with a saturated solution of corrosive sublimate, but bilirubin is changed into biliverdin, and the fæces turn green. The hydrobilirubin is the result of putrefactive processes in the intestine. It is hardly ever found in the breast-fed, because of the rarity of putrefactive intestinal changes. On the other hand Schikora has found it in the stools of healthy artificially-fed children. Some infants, overfed with cow's milk, but undernourished, pass almost white stools, like those of acholia. These stools are not acholic, but are the result of perverted liver function or change in the bile pigments. An abnormal reduction takes place, whereby bilirubin is converted into a colourless urobilinogen instead of into urobilin. With an acid solution of dimethylamido-azobenzaldehyde urobilinogen produces an intense red colour. Probably urobilinogen is derived from hydrobilirubin, forming a colourless compound in an alkaline solution.

